# Efficacy of Plant Extract‐Based Solutions Compared to Chlorhexidine and Miconazole Shampoo for the Treatment of Superficial Pyoderma in Dogs

**DOI:** 10.1002/vms3.70075

**Published:** 2024-12-02

**Authors:** Galia Sheinberg Waisburd, Camilo Romero Núñez, Alberto Martin Cordero, Rafael Heredia Cárdenas, Ariadna Flores Ortega

**Affiliations:** ^1^ Dermatologia Especializada Centro Veterinario México Ciudad de México México; ^2^ Dermavet Hospital Veterinario José de la Luz Blanco Ciudad de México México; ^3^ VETDERM: Dermatología Veterinaria Especializada Guadalajara Jalisco México; ^4^ Centro Universitario UAEM Amecameca Universidad Autónoma del Estado de México Amecameca de Juárez Estado de México México

**Keywords:** dogs, essential fatty acids, essential oils, mousse, natural formulations, shampoo, superficial pyoderma

## Abstract

**Background:**

Canine superficial pyoderma (CSP) is common. Antibiotic and potential antiseptic resistance is a rising concern; natural topicals may provide valuable alternative options.

**Objectives:**

Compare natural topicals containing essential oils, plant‐extracted essential fatty acids and N‐acetylcysteine to a medicated shampoo with chlorhexidine, miconazole and micronized silver in treating CSP.

**Animals:**

Thirty dogs diagnosed with CSP, divided into three groups as outlined below.

**Materials:**

Group 1 bathed using a shampoo (PYOclean Shampoo) twice weekly and application of a rinse‐free mousse (PYOclean Mousse) once daily, Group 2 bathed using the same shampoo as above twice weekly and application of a spray (PYOclean Spray) once daily, and Group 3 bathed using a medicated shampoo (Biohex Shampoo) twice weekly.

**Methods:**

Cytology, lesion and pruritus visual analogue (PVAS) scores were evaluated on Days (D) 0, 7 and 15.

**Results:**

All three groups showed decreased polymorphonuclear cells (PMN) on D15. The reduction was significant, and more significant in Groups 1 and 2 (90.48% and 98.93%) compared to Group 3 (85.96%); extracellular cocci (cocci EC) significantly decreased by 92.8% and 93.38% in Groups 1 and 2, whereas decreased by 81% in Group 3 on D15. The number of yeasts on D15 decreased by 90% in Group 3 and 82% and 92%, respectively, in Groups 1 and 2. Lesion scores decreased on D15 by 90.61% and 87.95% in Groups 1 and 2 and by 62.94% in Group 3. PVAS decreased on D15 by 95.78% and 96.75%, respectively, in Groups 1 and 2 and by 69.88% in Group 3.

**Conclusion and clinical importance:**

Efficacy of three natural topicals in treating dogs with superficial pyoderma without adding any systemic antimicrobial drugs was demonstrated. Such solutions can be complementary to medications such as chlorhexidine and miconazole in addressing CSP.

## Graphical abstract

Thirty dogs with CSP. Group 1 a shampoo (PYOcleanShampoo) twice weekly and a rinse‐free mousse (PYOclean Mousse) once daily. Group 2 the same shampoo as above twice weekly and a spray (PYOclean Spray) once daily. Group 3 a medicated shampoo (Biohex Shampoo) twice weekly. Groups 1 and 2 (90.48% and 98.93%) compared to Group 3 (85.96%). Efficacy of three natural topicals in treating dogs with superficial pyoderma without adding any systemic antimicrobial drugs was demonstrated.

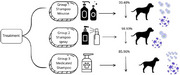

## 1 Introduction

Canine superficial pyoderma (CSP) represents a common and frequently recurrent condition in dogs. *Staphylococcus pseudintermedius*, the causative organism in most patients (Bäumer et al. 2017), is a commensal bacterium that resides on dogs’ mucosa and skin and acts as an opportunistic pathogen, sometimes with bacilli and yeasts. Superficial pyoderma is associated with interaction among genetic, environmental and immunological factors (Bajwa 2016). The use of systemic antimicrobials has been a standard treatment for this disease (Hillier et al. 2014). However, rising antibiotic resistance represents an increasingly complex challenge for veterinarians, and the importance of using topical antiseptic therapy as an alternative treatment has been stressed (Bond and Loeffler 2012). Due to increasing antimicrobial resistance rates in bacterial isolates, there is an urgent need for efficient complementary or alternative treatments (Bäumer, Jacobs, and Tamamoto‐Mochizuki 2020). The latest clinical guidelines for superficial bacterial folliculitis in dogs recommend focussing more on topicals while avoiding excessive use of systemic antibiotics to mitigate bacterial resistance development (Bond and Loeffler 2012). Products traditionally used for pyoderma management are chlorhexidine shampoos alone or in combination with miconazole or ethylenediaminetetraacetic acid (tris‐EDTA) (Clark et al. 2015). However, topical therapy with alternative treatments is increasingly studied and may help prevent the selection of multi‐drug–resistant staphylococci (Clark et al. 2016). The uses of topical products containing essential oils, plant‐extracted essential fatty acids and other natural compounds with antimicrobial or antibiofilm properties (such as manuka essential oil and *N*‐acetylcysteine) have been tested in pyoderma and cases of canine external otitis with positive results (Song et al. 2020). Natural ingredient‐based products can help speed up pyoderma resolution and may allow a shorter antimicrobial treatment time (Bensignor, Fabriès, and Bailleux 2016). This study aimed to compare the efficacy of natural topical solutions containing essential oils and plant extracts to a medicated shampoo containing chlorhexidine, miconazole, ceramides and micronized silver in treating CSP.

## 2 Materials and Methods

### 2.1 Animals

Thirty dogs of all breeds, in a range of 5–12 years, with weights of 7–50 kg, were otherwise healthy outside of their CSP, and females and males with superficial pyoderma and cytological evidence of bacterial infection were selected; they had not been treated topically or systemically for 2 weeks prior to the start of the study. The owners signed an informed consent form accepting the treatment.

### 2.2 Treatment

The type of study used is multi‐centre. The dogs were allocated randomly into 3 groups of 10.

Group 1: Twice weekly bathing with PYOclean Shampoo (plant‐extracted essential fatty acids of omega‐3, omega‐6 and green apple lipoamino acids, honey, propolis and hemp seed oil known to have antimicrobial properties; Dermoscent, Castres, France) and application of a daily rinse‐free foam of PYOclean Mousse (plant‐extracted essential fatty acids, manuka and lavandin essential oils, honey and *N*‐acetylcysteine; Dermoscent, Castres, France).

Group 2: Twice weekly bathing with PYOclean Shampoo and daily application of PYOclean Spray (plant‐extracted essential fatty acids, lavandind manuka essential oils, *N*‐acetylcysteine; Dermoscent, Castres, France).

Group 3: Twice weekly, baths were performed by a veterinarian with Biohex shampoo (chlorhexidine gluconate 2% and miconazole nitrate 2%, purified water, decyl polyglucoside, cetrimonium chloride, sodium olefin sulphonate, cocamidopropyl betaine, octoxynol 10, Laureth‐12, PEG‐120 methyl glucose dioleate, Disteareth‐75 IPDI, Glycereth‐7, ethylene glycol distearate, kiwi fragrance, Syncrystal silk silver, Polysorbate‐80, sodium metabisulfite and *N*‐octadecanoylphytosphingosine) (Ceramide III, Microsilver, sodium hydroxide; Vetbiotek, FL, USA). Treatments were applied according to the manufacturer's recommendation and shaken well before use. The hair coat was wet with warm water, and small quantities of shampoo were applied from the base of the neck to the base of the tail. The shampoo was massaged over the pet's body to ensure good contact with the skin and then allowed to remain on hair for 5–10 min. After this, the pet was rinsed thoroughly with clean water and the instructions repeated.

### 2.3 Cytological Evaluations

All samples were taken by direct impression smear and stained with a Romanowsky stain. The slides were examined using the 100× oil immersion objective, and a minimum of 10 fields were evaluated. Cytological evaluations (Udenberg et al. 2004) were performed for the assessment of polymorphonuclear leukocytes (PMN), the presence of intracellular (IC) and extracellular (EC) cocci, bacilli and yeast on Days (D) 0, 7 and 15. For each area and clinical lesion (pain, erythema, inflammation, ulcers, pigmentation and alopecia), a validated scoring system was used with values of 0 (none), 1 (light), 3 (moderate) or 6 (severe) assigned for seven different lesion sites (42 points maximum) in 14 body areas (maximum value of 588 points). The owner evaluated the pruritus severity using the pruritus visual analogue score, a previously validated scoring system. The VAS uses word descriptors on a scale and then is translated into a number out of 100 (Hill, Lau, and Rybnicek 2007).

### 2.4 Statistical Analysis

The data were recorded in a spreadsheet database, and Duncan's multiple range test, with an alpha of 0.05, was applied to compare the mean of the weekly assessments for each treatment. The initial value of all variables was used as a covariate for this model and analysed with the SAS 9.0 statistical software.

## 3 Results

### 3.1 Cytology

**FIGURE 1:**
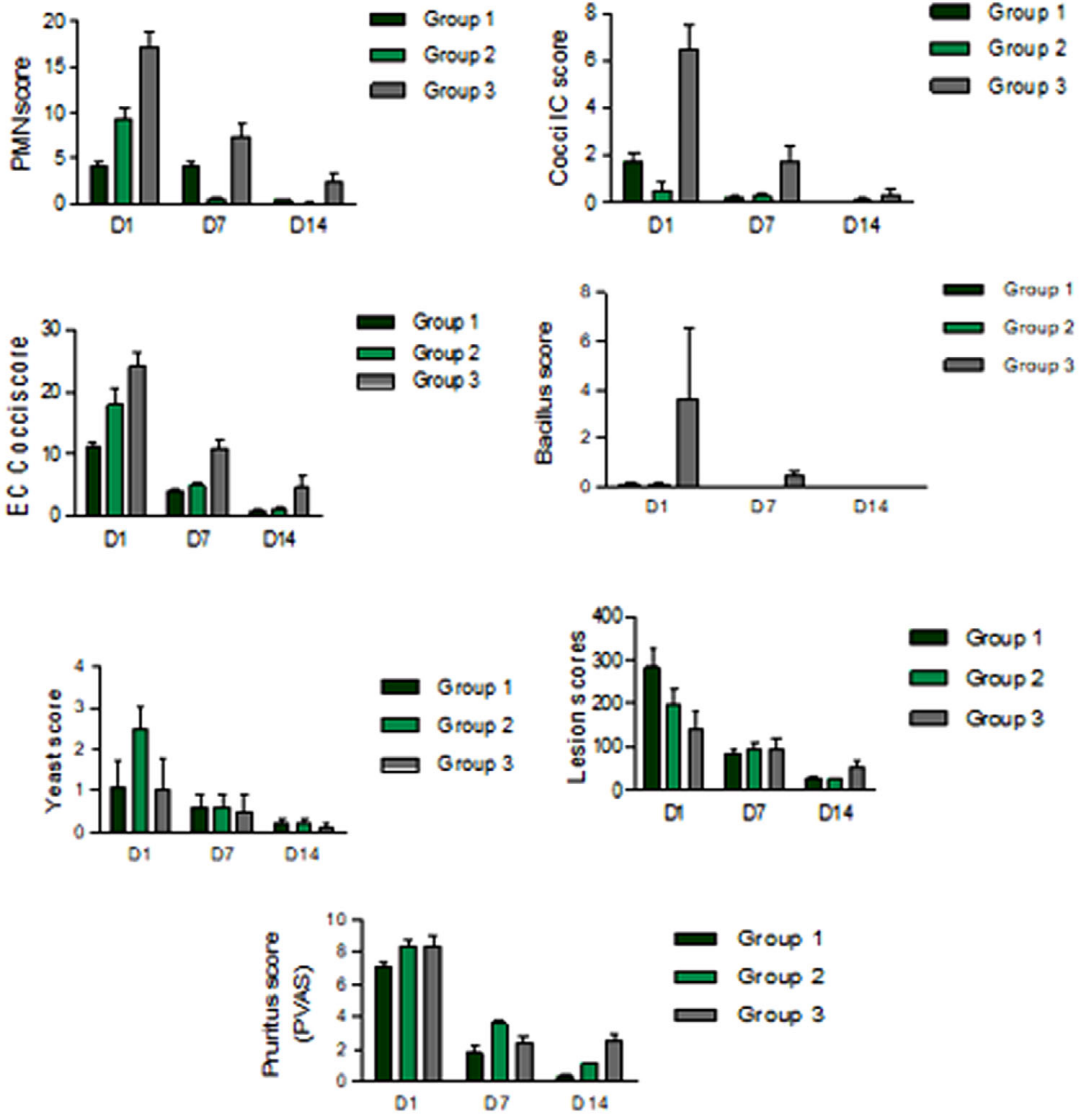
Behaviour of the variables analysed during 15 days of treatment. EC = cocci extracellular, EEM = standard error of the mean; IC = intracellular.

Table 1 presents the results of the variables evaluated in this study in detail. The mean PMN on D15 of each of the three treatments was significantly reduced (from 85.96% to 98.92%). However, the reduction was statistically greater in Groups 1 and 2 (98.93% and 90.48%) compared to Group 3 (Biohex, 85.97%) (*p* < 0.05). On D7, the number of IC cocci was reduced in the three groups, decreasing by 88.24% in Group 1 (mousse), 40% in Group 2 (spray) and 73.85% in Group 3 (Biohex) (Figure 1). All three treatments at the last evaluation on D15 were comparable without significant difference.

**TABLE 1:** Comparison of the effect between treatments of the presence of polymorphonuclear cell (PMN), cocci intracellular (IC), cocci extracellular (EC), bacillus, yeast, lesion score and itch scale.

	Treatments	Start of treatment	Day 7	Day 15
**PMN (20)**	Group 1	4.20b	4.10b 2.39%	0.40b 90.48%
	Group 2	9.30c	0.60c 93.55%	0.10b 98.93%
	Group 3	17.10a	7.40a 56.73%	2.40a 85.97%
	CV	64.89	98.20	202.35
	EEM	1.20	0.72	0.35
**Cocci IC (10)**	Group 1	1.70b	0.20b 88.24%	0a 100%
	Group 2	0.50b	0.30ab 40%	0.10a 80%
	Group 3	6.50a	1.70a 73.85%	0.30a 95.39%
	CV	113.59	199.17	428.50
	EEM	0.60	0.26	0.10
**Cocci EC (30)**	Group 1	11.10b	4.10b 63.07%	0.80b 92.8%
	Group 2	18.10a	5.0b 72.38%	1.20ab 93.38%
	Group 3	24.20a	10.90a 54.96%	4.60a 81%
	CV	45.33	62.07	167.28
	EEM	1.47	0.75	0.67
**Bacillus (30)**	Group 1	0.10a	0a 100%	0a 100%
	Group 2	0.10a	0a 100%	0a 100%
	Group 3	3.60a	0.40a 88.89%	0a 100%
	CV	437.32	380.56	—
	EEM	0.01	0.09	0
**Yeast (10)**	Group 1	1.10a	0.60a 45.46%	0.20a 81.82%
	Group 2	2.50a	0.60a 76%	0.20a 92%
	Group 3	1.00a	0.50a 50%	0.10a 90%
	CV	137.79	183.53	227.42
	EEM	0.38	0.18	0.06
**Lesion**	Group 1	283.20a	84.20a 70.27%	26.60a 90.61%
	Group 2	199.10a	94.60a 52.49%	24.00a 87.95%
	Group 3	143.0a	93.60a 34.55%	53.00a 62.94%
	CV	66.80	63.91	83.21
	EEM	25.42	10.59	5.24
**Pruritus**	Group 1	7.10a	1.80b 74.65%	0.30b 95.78%
	Group 2	8.30a	3.60a 56.63%	1.10b 86.75%
	Group 3	8.30a	2.40b 71.09%	2.50a 69.88%
	CV	21.63	50.09	97.19
	EEM	0.31	0.23	0.23

*Note*: Different letters (a, b) in column present significant difference, Group 1 = shampoo + mousse, Group 2 = shampoo + spray, Group 3 = Biohex, % = percentage of reduction, alpha = 0.05.

Abbreviations: CV = coefficient of variation; EEM = standard error of the mean.

**FIGURE 2:**
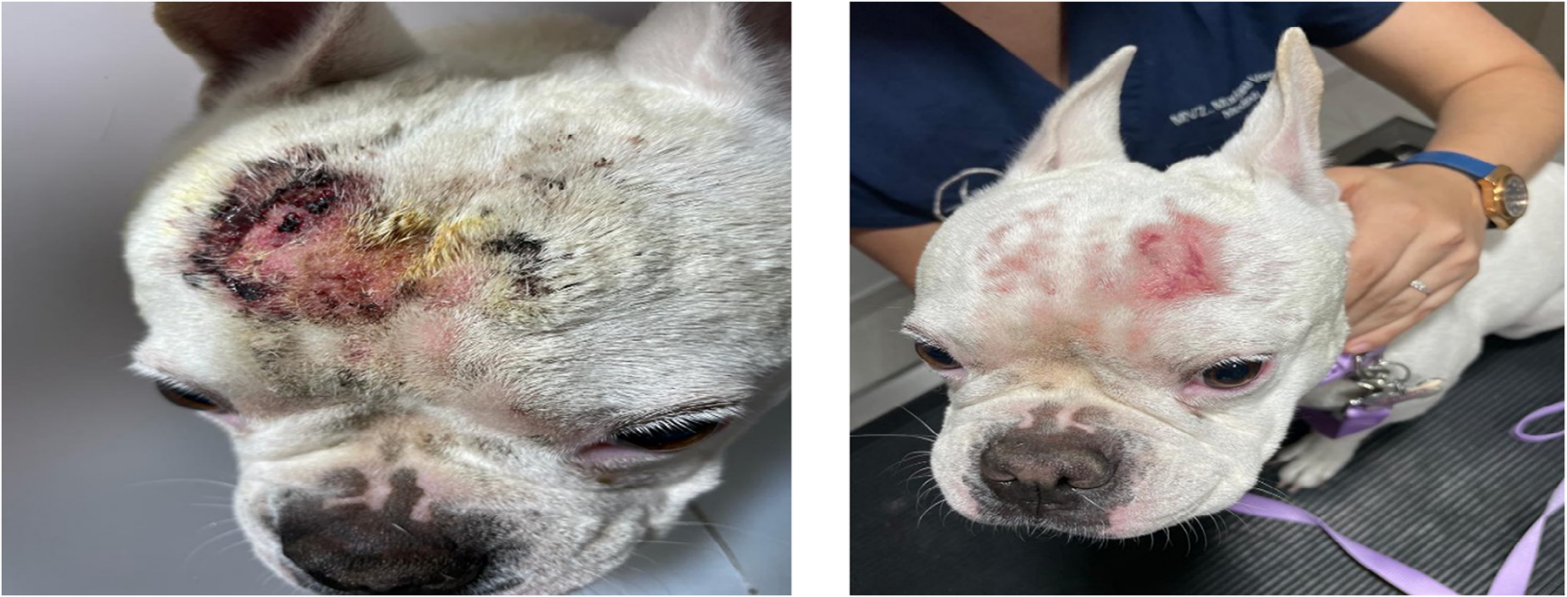
The lesion scores were significantly reduced in Group 1.

**FIGURE 3:**
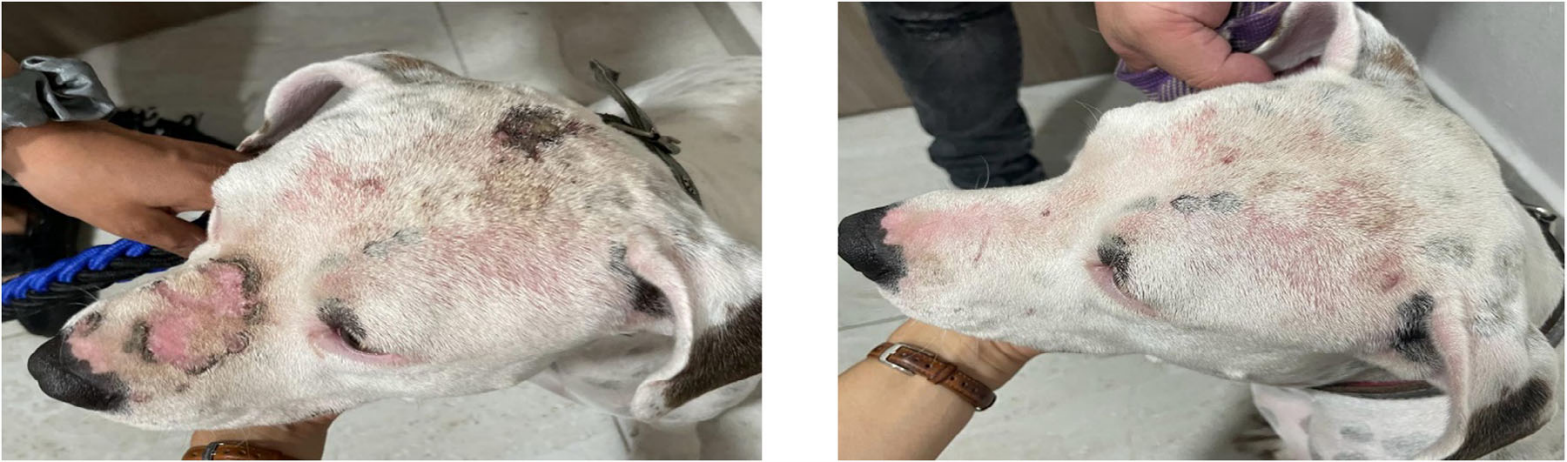
The lesion scores were significantly reduced in Group 2.

**FIGURE 4:**
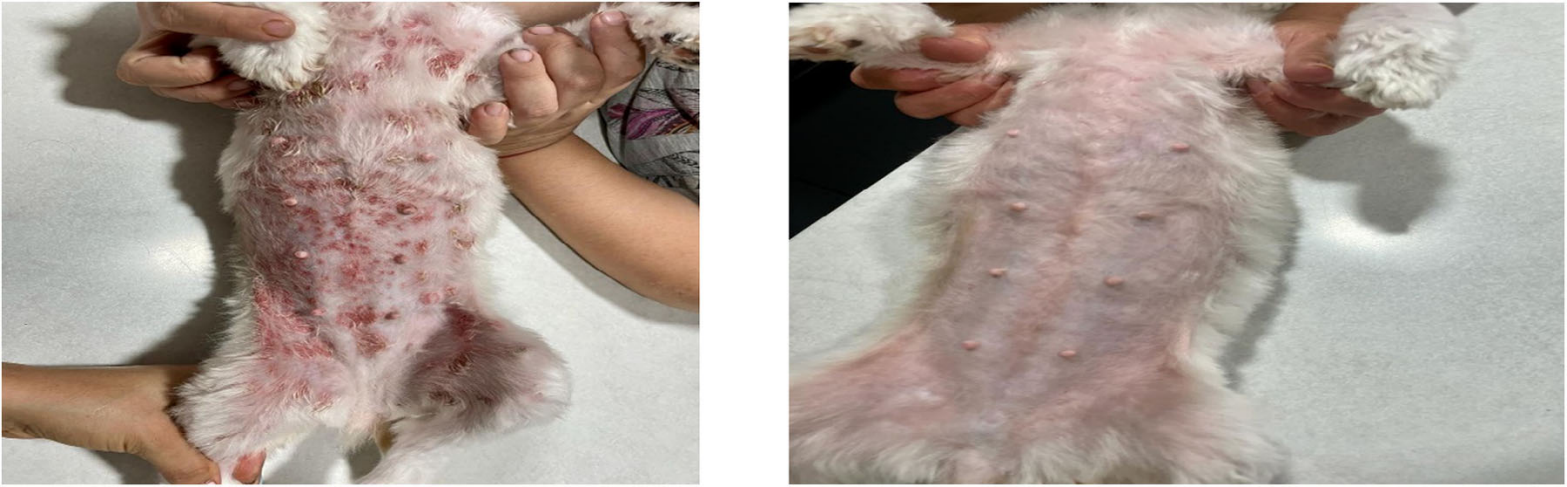
The lesion scores were significantly reduced in Group 3.

The EC cocci decreased on D7 by a greater percentage in Groups 1 and 2 (72.38% and 63.07%, respectively) compared to Group 3 (Biohex, reduction of 45.96%). On D15, EC cocci were reduced by 92.8% in Group 1, 93.38% in Group 2 and 81% in Group 3 (Biohex), with no significant difference among the groups Figure 2, 3, 4.

Bacilli were detected only on a few dogs, and all decreased by 100% on D15 without significant difference among the three groups.

The number of yeasts was comparable at the end of the study among all three groups without a statistical difference: The reduction was 90% for Biohex, 81.82% for Group 1 with the mousse and 92% for Group 2 with the spray.

### 3.2 Clinical Evaluation

The lesion scores were significantly reduced in all groups, showing no significant difference at the end of the study on D15. However, a greater reduction in Group 1 was observed on D7 by 70.27% and 90.61% on D15 compared to Groups 2 and 3.

The pruritus scale showed a significant decrease (*p* < 0.05) on D7 in all three groups: 71.09% with Biohex, 74.65% in Group 1 and 56.63% in Group 2, with moreover a significant difference (*p* < 0.05) between Groups 3 and 1. On D15, Group 3 had a similar reduction score of 69.88% as its performance on D7 (71.09%), whereas the reduction scores from both Groups 1 and 2 were, respectively, higher by 95.78% and 86.75%, with a significant difference compared to Group 3 (*p* < 0.05 for Group 1 and *p* < 0.001 for Group 2).

## 4 Discussion

The present study results have demonstrated the efficacy of natural topical products in the resolution of CSP without systemic or topical antimicrobial treatments. This research showed that treatments based on natural extracts have clinical and cytological benefits within 2 weeks of treatment. Moreover, the efficacy related to post‐bathing additional products used daily in Groups 1 and 2 is different from those described by Hillier et al. (2014), indicating that CSP would typically require 3–4 weeks of antibiotic treatment. The cytological analysis showed a significant decrease in PMN numbers on D15 in all three groups in this study. However, the reduction percentage was higher in Groups 1 and 2 than in Group 3, with statistical significance. Such results are in‐line with the findings of Song et al. (2013), in which manuka oil demonstrated anti‐biofilm and anti‐staphylococcal properties in vitro and was also proven functional against pyoderma. The IC cocci showed a marked decrease with each of the three treatments on D15 (*p* < 0.05), without a significant difference among the three. However, EC cocci decreased significantly on D7 with both Groups 1 and 2 compared to Group 3.

Such results are relevant because other research reports have shown that chlorhexidine is just as effective as systemic antimicrobial therapy with amoxicillin‐clavulanic acid (Borio et al. 2015). The repeated use of biocides such as chlorhexidine may be associated with skin irritation and skin dryness. There is also a potential for the selection of resistant pathogens related to resistance to mutations in the qacA gene that codes for antiseptic resistance protein (Horner, Mawer, and Wilcox 2012). Therefore, natural topical products offer a safe alternative to potentially irritating shampoos and, by reducing antibiotic use, the potential for selection of antimicrobial pathogens. In this study, although rare, all three treatments were effective. This study reveals that natural ingredients contained in the topical solutions of Groups 1 and 2 have antibacterial properties. *N*‐Acetylcysteine prevents biofilm formation and inhibits staphylococcal adhesion to the skin (Perez‐Giraldo et al. 1997). Essential oil of manuka is demonstrated in other studies to have high in vitro activity against Gram‐positive bacteria (Song et al. 2020). In this study, efficacy against cocci and yeast was noted.

There was no significant difference in lesion scores among the three groups. Nevertheless, during this study, Group 1 showed the highest reduction on both D7 and D15. On the pruritus scale, Groups 1 and 2 were more effective on D15 compared to Group 3. It is possible that daily rinse‐free treatment, either the mousse or the spray in Groups 1 and 2, could have contributed to better clinical performance when compared to the medicated shampoo alone.

In conclusion, this study demonstrated natural topicals' clinical and cytological efficacy in treating CSP without systemic antimicrobial drugs. Formulated with plant‐derived essential fatty acids, essential oils and other plant extracts such as *N*‐acetylcysteine with known antibacterial or antibiofilm properties, such natural topical products can be considered alternatives to the medicated shampoos containing chlorhexidine and miconazole typically used in treating CSP.

## Author Contributions

**Galia Sheinberg Waisburd**: validation, visualization, methodology, experimentation, supervision. **Camilo Romero Núñez**: conceptualization, resources, project administration, supervision, validation, experimentation, visualization, writing–review and editing. **Alberto Martin Cordero**: validation, visualization, methodology, experimentation, supervision. **Rafael Heredia Cárdenas**: data curation, formal analysis, software, roles/writing–original draft, visualization. **Ariadna Flores Ortega**: project administration, investigation, resources, supervision, validation, experimentation, roles/writing–original draft.

## Conflicts of Interest

The authors declare no conflicts of interest.

### Peer Review

The peer review history for this article is available at https://www.webofscience.com/api/gateway/wos/peer-review/10.1002/vms3.70075.

## Data Availability

The data that support the findings of this study are available from the corresponding author upon reasonable request.
